# Validation of a Framework for Measuring Hospital Disaster Resilience Using Factor Analysis

**DOI:** 10.3390/ijerph110606335

**Published:** 2014-06-18

**Authors:** Shuang Zhong, Michele Clark, Xiang-Yu Hou, Yuli Zang, Gerard FitzGerald

**Affiliations:** 1 Center for Emergency and Disaster Management, School of Public Health and Social Work, Institute of Health and Biomedical Innovation, Queensland University of Technology, Brisbane, Queensland 4059, Australia, E-Mail: x.hou@qut.edu.au; 2 Center for Health Management and Policy, Shandong University, Jinan, Shandong 250012, China; 3 School of Clinical Sciences, Queensland University of Technology, Brisbane, Queensland 4059, Australia; E-Mail: mj.clark@qut.edu.au; 4 School of Nursing, Shandong University, #44 Wenhua West Road, Jinan, Shandong 250012, China; E-Mail: yulizh@sdu.edu.cn

**Keywords:** disaster, evaluation framework, factor analysis, hospital resilience, preparedness

## Abstract

Hospital disaster resilience can be defined as “the ability of hospitals to resist, absorb, and respond to the shock of disasters while maintaining and surging essential health services, and then to recover to its original state or adapt to a new one.” This article aims to provide a framework which can be used to comprehensively measure hospital disaster resilience. An evaluation framework for assessing hospital resilience was initially proposed through a systematic literature review and Modified-Delphi consultation. Eight key domains were identified: hospital safety, command, communication and cooperation system, disaster plan, resource stockpile, staff capability, disaster training and drills, emergency services and surge capability, and recovery and adaptation. The data for this study were collected from 41 tertiary hospitals in Shandong Province in China, using a specially designed questionnaire. Factor analysis was conducted to determine the underpinning structure of the framework. It identified a four-factor structure of hospital resilience, namely, emergency medical response capability (F_1_), disaster management mechanisms (F_2_), hospital infrastructural safety (F_3_), and disaster resources (F_4_). These factors displayed good internal consistency. The overall level of hospital disaster resilience (F) was calculated using the scoring model: F = 0.615F_1_ + 0.202F_2_ + 0.103F_3_ + 0.080F_4_. This validated framework provides a new way to operationalise the concept of hospital resilience, and it is also a foundation for the further development of the measurement instrument in future studies.

## 1. Introduction

Disasters including extreme weather events, natural disasters, bioterrorism, and pandemics are having an increased global impact [1]. Hospitals play an important role during disasters, as they provide “lifeline” services to reduce disaster associated mortality and mobility, and thus minimize the impact of disasters on the community [2,3,4,5]. Efficient hospital disaster management is considered an essential way for hospitals to supply continuous health services during disasters, even if the hospitals are directly affected by the disaster [6,7]. Hospital resilience is an emerging concept that has been added into the hospital disaster management context [2,8,9,10,11,12]. It can be defined as “the ability of hospitals to resist, absorb, and respond to the shock of disasters while maintaining and surging essential health services (e.g., on-site rescue, pre-hospital care, emergency health treatment, critical care, decontamination and isolation), and then to recover to its original state or adapt to a new one.” [10]. It implies a comprehensive perspective of a hospital’s ability to cope with disasters, including inherent strength (ability to resist and absorb disasters) and adaptive flexibility (strategies to maintaining and surging essential health services and adaptation for future disasters) [13,14,15]. There are four criteria of disaster resilience which can be adapted into hospitals, namely, robustness, redundancy, resourcefulness, and rapidity [5,8,9,12]. To be more specific, hospital resilience aims to improve hospital pre-event strength, thus promoting the rapidity of response and recovery, through redundant resources and resourceful strategies [10,16,17,18,19,20].

Defining and measuring hospital resilience has been the subject of recent research [2,5,8,9,10,12,21]. Most of these measures were derived from the engineering perspective, and used mathematical calculations; there is little empirical evidence of a hospital resilience evaluation instrument that can be used easily by hospital managers and health administrators. Recently, the primary focus of studies in the area of health disaster management has been on the evaluation of disaster preparedness [22,23,24,25,26,27]. However, such disaster preparedness studies tend to adopt a particular perspective of the pre-disaster stage, instead of examining the institution’s overall ability to confront disasters. For example, previous research emphasizes the structural components (e.g., human, equipment, resources), but devalues the importance of functional components, such as safety infrastructure, and medical response and surge capacity [10,28,29,30]. Another weakness of most of the existing frameworks is that they are not validated by empirical data [10].

Most studies view hospital disaster ability separately, using concepts such as hospital safety, preparedness, response, recovery, business continuity, surge capacity, and other specific abilities [26,27,28,31,32]. These concepts do not achieve consensus around a comprehensive framework for evaluating a hospital’s ability through all phases of the disaster management [28,29]. Thus, there is value in developing a comprehensive instrument, which can be used as an evaluation tool by hospital practitioners, managers and government administrators to understand and assess the overall ability of hospitals to cope with disasters. The concept of disaster resilience (which is a measure of return of functions) is a new but important concept that is useful to help devise this instrument [12].

This article aims to develop and validate a framework for hospital resilience, and assess its validity and reliability using empirical data from tertiary hospitals. It has four objectives: (1) to develop a preliminary evaluation framework of hospital resilience; (2) to extract key component factors from the primary domains of the framework for further conceptualization and measurement of hospital resilience; (3) to examine the construct validity and internal consistency of this framework; and (4) to establish scoring models to measure the level of hospital disaster resilience.

## 2. Methods

### 2.1. Study Design

This study was a cross-sectional study of tertiary hospitals in Shandong Province in China. The survey was conducted between January 2013 and June 2013. Ethical approval to conduct the research was obtained from the Queensland University of Technology (approval No. 1200000170) and written informed consent was obtained from each participating hospital.

### 2.2. Study Setting and Sample

In China a tertiary hospital is defined as a health facility covering a large administrative area and capable of providing comprehensive and specialized medical care. For tertiary hospitals, the total number of beds should be more than 500, with physician and nurse numbers being more than 1.03 and 0.4 per bed, respectively. Tertiary hospitals can be further classified into three subgroups: Grade A, Grade B, and Grade C. Such classification was derived from the national hospital evaluation according to each hospital’s comprehensive scores (the total score is 1000) aggregated from respective perspectives, such as service levels, size, medical missions, medical technology, medical equipment, management, and medical quality [33,34]. Tertiary A hospitals have achieved higher comprehensive scores than tertiary B and tertiary C hospitals. Thus, tertiary A hospitals are supposed to be the most advanced hospitals in an area [34]. A total of 50 tertiary hospitals in the Shandong Province were selected using stratified random sampling, according to their subgroups (*i.e.*, Grade A, B, or C). The hospital selection used the contact list obtained from the Provincial health department. A total of 41 fully completed questionnaires were received, representing an 82.0% response rate. The response quantity accounted for approximately half (53.9%) of the total quantity of tertiary hospitals in this province.

### 2.3. Study Protocol

A preliminary framework for assessing hospital resilience was proposed through a systematic literature review [10]. This review used four criteria of disaster resilience (*i.e.*, robustness, resourcefulness, redundancy and rapidness) as inclusion criteria for selecting key domains and indicators from the included studies [10]. More details regarding such selection can be found in this review [10]. The potential indicators in this framework were then developed and validated by a Modified-Delphi study undertaken in China. This study was used to collect experts’ opinions regarding key indicators of hospital resilience [35]. From the previous systematic approach, a preliminary evaluation framework was established. Within this framework, eight key domains were identified that can be used to reflect the level of hospital resilience. Then, based on the preliminary framework, a hospital survey questionnaire was designed to collect the data. The resultant questionnaire consisted of nine sections, with more than 100 items in total.

As the study was conducted in China, a Chinese language questionnaire was used to capture the responses; the instrument was subsequently translated back into English for the final reporting. Brislin’s model of translation was used to ensure the accuracy and appropriateness of the translation of the questionnaires [36]. The survey questionnaire was forward-translated and then back-translated between the source language (English) and the target language (Chinese), by two independent translators (Shuang Zhong, Yuli Zang). The English version of the questionnaire can be found in the Supplementary Information.

The feasibility and suitability of the questionnaire were evaluated and modified by a pilot study at three hospitals. The questionnaire, accompanied by an official letter from the Provincial Health Department outlining the importance of the survey, was sent to each of the sample hospitals by both email and post. Each hospital was asked to designate a director in the management department to be responsible for distributing and coordinating the completion of the questionnaire. The questionnaires were completed and signed-off by suitably knowledgeable staff from relevant departments within the hospital. These departments included the emergency department, human resource management, pharmacy, resource logistics, and facility maintenance department. Each returned questionnaire was reviewed for its completeness and consistency. For those questionnaires which were incomplete and/or contained inconsistent responses follow-up telephone calls were made to ensure completeness and consistency.

### 2.4. Measurements

The first section of the questionnaire includes demographic information, while sections 2–9 represent the eight key domains that were identified in the preliminary evaluation framework for hospital disaster resilience, namely: hospital safety standard and procedures, emergency command, communication and cooperation system, disaster plan, disaster resource stockpile and logistics management, emergency staff capability, emergency services and surge capability, training and drills, and recovery and adaptation strategies. Most questions are in the format of binary variables, and can be answered by “yes” or “no” (e.g., is there any disaster plan?). Some questions are in the format of numeric variables, and can be answered by quantity numbers (e.g., how many staff are there in the rescue teams?).

The numeric indicators were mainly used for a qualitative description of hospital status [37], while the primary binary variables were included into the calculation of the resilience index. For the binary variables, the options of “yes” or “no” were assigned the score of “1” or “0”, respectively. Then, the scores of each domain were calculated by adding together the score of all the relevant questions. A total score was calculated by summing the scores across all eight domains, which is a proxy for measuring overall disaster resilience in an institution. The higher the total score, the better the hospital’s disaster resilience.

### 2.5. Data Analysis

The data from the returned questionnaires were transferred into a Microsoft Office Access 2007 database. The data were checked, cleaned, and analyzed using SPSS Statistics [38]. The study used several steps and methodologies during the data analysis. Firstly, to test the construct validity, factor analysis was chosen to analyze the empirical data. Factor analysis can be used to identify the underlying component factors between the measured variables and the latent constructs, thereby allowing the formation and refinement of the theory. It also can provide evidence of construct validity evidence of self-reporting questionnaires [39]. The steps of the factor analytical process included assessing the suitability of using factor analysis, correlation matrices, factor extraction, choosing the number of factors to retain, factor rotation, component score coefficient matrix, and factor interpretation [40]. Secondly, the discriminate validity of the construct was tested. It assumed that the level of the hospital’s ability to cope with a disaster is associated with the level of the hospital’s categorization [25,41]. For instance, it was expected that tertiary A hospitals would report a generally higher level of resilience than tertiary B hospitals, as the missions of regional disaster centers are primarily assigned to tertiary A hospitals. The scores of each factor, according to hospital categorization, were compared using independent sample t-tests. Thirdly, a self-assessment scale was developed based on the items in the questionnaire to categorize hospitals into different resilience levels. Also a model for calculating the overall level of disaster resilience among hospitals was established through each component factor being weighted. Finally, to test the internal consistency of key domains, the Cronbach’s coefficient alpha analysis was performed [42]. In addition, the current status of the sample was investigated using descriptive analysis, and was reported in a published article [37].

## 3. Results

### 3.1. Inspection

The value of the Kaiser-Meyer-Olkin Measure of Sampling Adequacy (KMO) in the current study is 0.792, which is over the threshold of 0.7. The result of Bartlett’s Test (*p* < 0.01) revealed that the eight primary domains are not independent, and have a high level of correlation. Thus, factor analysis is suitable to be used to extract component factors in the study [42].

### 3.2. Extraction of Component Factors

The factor solution was determined by the number of factors generated with eigenvalues greater than 1, as well as by theoretical considerations. The domain scores were used as the independent variable for component extraction. Four factors had an eigenvalue greater than one that could be extracted to be representative of all the domains. The 4-factor solution was compared to solutions with larger and smaller numbers of factors. The 4-factors solution appeared to be more meaningful theoretically than the smaller or larger factor solutions, which were difficult to interpret. As illustrated in Table 1, the 4-factor solution accounts for about 86.9% of the cumulative variance, and all the domains loaded highly (at least 77.0%).

**Table 1:** Total variance explained.

Domains	Extraction	Eigenvalues	% of Variance	Cumulative %
1. Emergency command, communication and cooperation system	0.770	2.201	52.653	52.653
2. Disaster plans	0.874	2.121	17.685	70.338
3. Disaster stockpiles and management	0.924	1.199	9.050	79.388
4. Emergency staff	0.895	1.035	7.552	86.940
5. Emergency training and drills	0.789	0.604	4.906	91.846
6. Emergency services and surge capacity	0.911	0.311	3.883	95.729
7. Hospital safety standard and procedures	0.973	0.181	2.259	97.989
8. Recovery and adaptation strategies	0.821	0.161	2.011	100.000

Note: Extraction method: principal component analysis.

### 3.3. Rotation

The relationship between the initial domains and the extracted components was not obvious. Thus the varimax rotation was used to make the extracted components more decentralized, and their relationship clearer and easier to explain [42]. These principal components can be explained with their significance; therefore, in this study these principal components are referred to as component factors.

According to the Rotated Component Matrix, each of the four factors extracted the majority of information from different domains (the percentage of the extracted information from domains is expressed in the parentheses). The first factor contains information mainly from three domains: essential service maintenance and surge capacity (0.901), emergency staff capability (0.891), and training and drills (0.625). It was assumed that these three domains reflect or impact upon a hospital’s medical response performance during disasters, and thus is named “emergency medical response capability”. The second factor was closely related to the domains of disaster plans (0.611), crisis leadership and cooperation (0.762), and recovery and adaptation strategies (0.878), and reflects “hospital disaster management mechanisms”. The third factor was mainly representative of the domain of disaster stockpiles (0.896), which focuses on a hospital’s “disaster resources”. The fourth factor includes hospital infrastructural strength and safety strategies to cope with disasters, and was representative of “hospital safety and vulnerability” (0.951). Hence, the four factors were identified and named: emergency medical response capability (F_1_), disaster management mechanisms (F_2_), infrastructure safety (F_3_), and disaster resources (F_4_). They explain most of the overall level of the hospital’s disaster resilience.

### 3.4. The Construction of the Framework

The results of the factor analysis are largely consistent with the original, theoretically developed framework, established from the literature review and the Modified-Delphi Method. In the validated framework the 43 measurable indicators were divided into four factors for conceptual understanding of hospital resilience. A summary of these results are presented in Table 2.

**Table 2:** Validated framework for evaluating hospital disaster resilience (adapted by the authors from [10]).

Construct of Measurable Items
**Factor 1: Emergency Medicine Response Capability**
**Domain 1: Emergency Services and Surge Capacity (Include On-site Rescue, Hospital Treatment, and Surge Capacity)**
The quantity and types of equipment for on-site rescue Equipment for referral and counter-referral (*i.e.*, transferring patients from or between places of medical treatment ) of special patients Communication equipment for on-site rescue Emergency medical treatment places and capability for different types of diseases The types and quantity of hospital emergency medical treatment equipment Hospital internal rapid assessment (e.g., evaluate the loss of manpower, beds, equipment after disasters) Hospital mass-casualty triage protocol The procedures to identify, prioritize, and maintain essential functions (e.g., cancellation of elective admissions, early discharge of patients, making new medical quality standards during disasters, extra protection for vulnerable population) Surge capacity of emergency beds (the surge rapidity, proportion and strategies for emergency space, emergency beds) Surge capacity of emergency resources (the surge rapidity, proportion and strategies for emergency equipment, medication and resource) Surge capacity of emergency staff (the surge rapidity, proportion and strategies for emergency staff)
**Domain 2: Emergency Staff Capability (Refer to Emergency Staff Specialties, Qualifications and Supporting Strategies)**
12. Staff composition of emergency expert group for different types of events 13. Staff composition of emergency expatriate team (*i.e.*, send experts or emergency staff to support on-site rescue, or local hospital treatment) for different types of events 14. Staff protection and resilient support (e.g., insurance, immunization, psychosocial support)
**Domain 3: Emergency Training and Drills (Disaster Training and Drills Involved in Hospital Daily Work)**
15. Training for different incident types (e.g., natural disasters, epidemics, and bioterrorism) 16. The percentage of key staff for training 17. The contents of training (e.g., triage, emergency health treatment, disaster management knowledge) 18. The frequency of training 19. Different incident types for drills 20. The methods for implementing drills (e.g., desktop drill, community-wide drill) 21. The frequency of drills
**Factor 2: Disaster Management Mechanisms**
**Domain 4: Emergency Command, Communication and Cooperation System**
22. Incident command system for disaster management (e.g., establishment of the disaster committee or responsible department) 23. The crisis cooperation within hospitals 24. The crisis communication and cooperation with community facilities (e.g., other hospital facilities, government offices, and police, fire department, the media, and the public)
**Domain 5: Disaster Plans (Plans to Prepare All-hazards Disasters in Advance)**
25. Plans for different kinds of disasters (for different single risk) 26. The staff coverage of disaster plans within hospitals 27. The period of evaluating and revising the plan 28. The plan initiation (e.g., the rapidity for staff, equipment can be in place when initiating the plan) 29. The extent to which the plan can be executed 30. Different and flexible responsive procedures for different disaster levels and phases (*i.e.*, classification response system for different levels and phases of disasters)
**Domain 6: Recovery and Adaptation Strategies (for Recovery and Improvement after Disasters)**
31. Hospital reconstruction and recovery mechanisms (e.g., responsible department, reconstruction support) 32. The strategies for community health recovery 33. The content of the after disaster evaluation report (e.g., vulnerability analysis, risk reassessment, capability analysis) 34. The adaptation strategies after disasters (*i.e.*, to adapt to a better state to cope with future disasters)
**Factor 3: Disaster Resources**
**Domain 7: Disaster Stockpiles and Logistics Management (Stockpile and Management of Emergency Supplies and Medications)**
35. Stock types and quantity for different emergency supplies (e.g., clean water, food, blood, emergency medical suppliers, portable medical equipment, ventilators and *etc.*) 36. The strategies for management of emergency resources (e.g., logistics and distribution, contracts with suppliers and other hospitals, adjusted standards for their usage) 37. Stock types and quantity for essential medications for various disasters (e.g., antimicrobial agents, cardiac medications, insulin, anti-hypertensive agents, IV fluids, *etc.*) 38. Strategies for management of medications (e.g., drug-distribution plans, drug management policy)
**Factor 4: Hospital Infrastructral Safety**
**Domain 8: Hospital Safety Standard and Procedures (Refer to Hospital Infrastructural Safety, Surveillance System, and Network Backup)**
39. The safety standard for hospital’s critical infrastructures to meet of high risks (e.g., for earthquakes, floods, fires, and isolation for infectious diseases) 40. Evaluation of hospital risks and vulnerabilities before disasters (e.g., hospital vulnerability assessment, risk assessment, the strategy to evacuate and protect existing patients) 41. The surveillance events (e.g., abnormity in admission diagnosis, surveillance of emergency room patients and death with unknown causes) 42. Analysis, report and sharing of surveillance information 43. The alternative hospital networks for emergency backup (e.g., power, water, oxygen and telecommunication)

Note: The key indicators were derived from the included studies [3,22,23,25,26,27,43,44,45,46].

### 3.5. Framework Validity Discrimination

A comparison of the scores (total score and mean score of each factor) among different categories of hospitals is shown in Table 3. As illustrated, the mean score of each factor of tertiary grade A hospitals is higher than that of tertiary grade B hospitals; the statistical difference is confirmed among all the factors. The mean scores of the four factors for the general hospitals are higher than that of specialized hospitals, except for the factor of hospital safety and vulnerability. However, statistical difference among the factors of emergency medical response (F_1_) and disaster resources (F_3_) was tested. The mean score of each factor of hospitals with the mission of “regional disaster rescue” is higher compared to hospitals without this mission. Statistical differences among most factors were tested. The overall scores for the tertiary A hospitals, general hospitals, and hospitals with the mission, are higher than that of tertiary B hospitals, specialized hospitals, and hospitals without the mission, respectively.

**Table 3:** Comparison of mean score (MS) and 95% of confidence interval of means (CI) of four factors of hospital resilience, using the hospital sample in Shandong, China, 2012.

Variables		No.	Component Factors				
			Emergency Response (F_1_)	Management Mechanisms (F_2_)	Safety (F_3_)	Resources (F_4_)	Overall Score (F)
			MS	MS	MS	MS	MS
			CI	CI	CI	CI	CI
Level	Tertiary A	27	29.26	12.89	8.07	2.26	52.48
			24.33, 34,19 *	11.85, 13.93 *	7.44, 8.70 *	1.98, 2.54 *	46.69, 58.27 *
	Tertiary B	14	24.29	7.86	6.93	1.29	40.36
			15.95, 32.62 *	6.58, 9.14 *	6.31, 7.55 *	0.63, 1.94 *	30.45, 50.27 *
Type	General	27	31.60	11.30	7.63	2.00	52.52
			26.23, 36.96 *	9.83, 12.76	7.09, 8.17	1.59, 2.41 *	45.63, 59.40 *
	Specialized	14	19.79	10.93	7.79	1.79	40.29
			14.96, 24.61 *	9.20, 12.66	6.72, 8.85	1.27, 2.30 *	34.08, 46.49 *
Disaster Mission	Assigned	13	37.31	13.92	8.23	2.38	61.85
			31.27, 43.34 *	12.26, 15.59 *	7.48, 8.98	2.08, 2.69 *	54.99, 68.70 *
	No mission	28	23.04	9.89	7.43	1.71	42.07
			18.34, 27.74 *	8.74, 11.05 *	6.81, 8.05	1.29, 2.13 *	36.42, 47.73 *
No. Category	Total	41	27.56	11.17	7.68	1.93	48.34

Notes: Emergency medical response capability, (highest score = 51); Emergency management mechanisms, (highest score = 17); hospital safety and vulnerability, (highest score = 9); disaster resources (highest score = 4); MS: Mean score CI: 95% confidence interval of means. * *p* < 0.05; Tested by non-parameter test (Mann-Whitney Test).

### 3.6. Scoring and Modelling

#### 3.6.1. Hospital Self-evaluation Scale Card (without Weighted)

When the questionnaire is used by hospitals for their self-assessment, the scale card as set out in Table 4, can be used to readily assess hospital vulnerabilities. There are 81 binary questions in the questionnaire that were included to reflect the key indicators in the evaluation framework. When all parts of the questionnaire were finished, the scores of these questions, “0” or “1”, can be summed. The highest score of each factor is illustrated at the bottom of the Table 3. The overall resilience score was summed up by adding the scores of the four factors (the highest score was 81). The quartile (25%, 75%) was used to categorize the levels of overall resilience score, and each dimension score. Such classification criteria were derived from the research approach of the Torrens Resilience Institute, in 2012, regarding the development of a scorecard toolkit to measure community disaster resilience [47]. As a result, if the overall score is over 60, the hospital was likely to be extremely resilient to disasters. If the overall score is less than 20, the hospital was likely to be extremely impacted upon in a disaster and/or have greater difficulty in recovering. Particular attention needed to be paid to the scores in the four components of resilience. If the individual score in one component tended to be in the low zone, this aspect of resilience likely indicated the highest priority for action. Thus, all scores can be very useful in highlighting those aspects of resilience that most need attention from hospital managers and relevant decision-makers. For instance, adapting the data in this study sample (Table 3) into the scale card (Table 4), the hospitals’ average overall resilience was found to be located in the “moderate zone” (score = 48.34). Among the four factors, only the score of hospital safety (F_3_) was located in the “high zone” of resilience (score = 7.68), which indicates a good level of resilience for that factor. The scores of the other three factors (F_1_, F_2_, and F_4_) were located in the “moderate zone”.

**Table 4:** Scale card for self-evaluation and categorization of the level of hospital resilience.

Factors	Low Zone	Moderate Zone	High Zone
Overall	25% (0–20)	26%–75% (21–60)	76%–100% (61–81)
Emergency medical response	25% (0–12)	26%–75% (13–38)	76%–100% (39–51)
Management mechanisms	25% (0–4)	26.2%–75% (5–12)	76%–100% (13–17)
Safety and vulnerability	25% (0–2)	26%–75% (3–6)	76%–100% (7–9)
Resources	25% (0–1)	26%–75% (1–3)	76%–100% (3–4)

Notes: Low zone (poor level of resilience); Moderate zone (moderate level of resilience); High zone (Resilient). The classification criteria of 25% and 75% were derived from the research approach of the Torrens Resilience Institute, in 2012 regarding the development of a scorecard toolkit to measure community disaster resilience [47].

#### 3.6.2. Scoring Model for Comparison and Rank of Hospitals in an Area (Weighted)

To compare or rank disaster resilience among hospitals, the weight of each factor was taken into consideration, as different factors make different contributions to the overall resilience [37].

The score for each factor for each of the sample hospitals was obtained by the use of regression analysis based on the Component Score Coefficient Matrix. Each factor score for the study hospital sample can be expressed using the following model [11]. Data is from Zhong, *et al.* 2014 [37]:

F_1_ = 0.524 X_1_ + 0.570 X_2_ + 0.191 X_3_ − 0.080 X_4_ − 0.220 X_5_ − 0.083 X_6_ − 0.147 X_7_ + 0.080 X_8_ F_2_ = 0.030 X_1_ − 0.315 X_2_ + 0.138 X_3_ + 0.443 X_4_ + 0.182 X_5_ + 0.659 X_6_ − 0.214 X_7_ − 0.211 X_8_ F_3_ = −0.253 X_1_ − 0.116 X_2_ + 0.093 X_3_ + 0.007 X_4_ + 0.364 X_5_ − 0.318 X_6_ + 0.936 X_7_ − 0.199 X_8_ F_4_ = −0.144 X_1_ + 0.288 X_2_ − 0.109 X_3_ − 0.119 X_4_ + 0.148 X_5_ − 0.167 X_6_ − 0.148 X_7_ + 0.972 X_8_ (1)

The weight of each factor was assigned by the proportion of the variance contribution of each principal to the cumulative variance contribution of the four primary factors (as in Table 1) [42]. Then the evaluation model of hospital disaster resilience was established and the overall level of hospital disaster resilience (F) can be calculated using the following model:

F = 0.615 F_1_ + 0.202 F_2_ + 0.103 F_3_ + 0.080 F_4_ (2)

The weight for emergency medical response capability was 0.615, for disaster management mechanisms 0.202, for hospital infrastructural safety 0.103 and for disaster resources 0.080. The disaster resilience score for each hospital in the study was calculated accordingly; their relative rank is listed in Table 5. Due to ethical issues and respect for confidentiality, the hospital’s name was replaced with a hospital ID.

**Table 5:** Overall score of disaster resilience (F) and their rank in the hospital sample (data from Zhong *et al.* 2014 [37]).

ID	F	Rank	ID	F	Rank	ID	F	Rank	ID	F	Rank
1	−0.554	32	12	0.482	11	23	1.120	1	34	0.733	7
2	−0.997	37	13	−0.195	26	24	−0.093	23	35	0.331	16
3	0.384	13	14	−0.970	36	25	0.095	19	36	0.733	8
4	−0.206	27	15	−1.189	41	26	−0.262	30	37	1.088	2
5	−0.750	35	16	−1.126	40	27	−0.085	22	38	0.932	3
6	−0.147	24	17	−1.005	38	28	0.774	6	39	−0.176	25
7	0.049	20	18	−1.116	39	29	0.623	9	40	0.360	14
8	−0.239	29	19	−0.04	21	30	−0.572	33	41	−0.729	34
9	0.262	17	20	0.8624	4	31	−0.235	28			
10	0.550	10	21	0.7885	5	32	0.412	12			
11	−0.33	31	22	0.3393	15	33	0.0990	18			

During the analysis, regression factor scores were standardized by construction. Thus, the illustrated scores were relative scores. A negative score means that the ratings of the level of resilience was lower than average. If a score was closer to the value of 0, it meant that the ratings of the level of overall resilience were closer to the average of the sample [48]. According to Table 5, there were 20 hospitals whose average comprehensive scores (F) were positive, which accounts for approximately 50% of the sample. The results indicate that hospital disaster resilience in the province was insufficient with considerable variation in the likely resilience of those hospitals which were sampled. Similarly, the score of each four factors (F_1_, F_2_, F_3_, and F_4_) can be calculated and ranked respectively, and thus can assist in identifying areas for highest priority in relation to strengthening resilience.

### 3.7. Internal Consistency

The results indicate that the four extracted component factors had a generally acceptable level of internal consistency (Cronbach’s Alpha Based on Standardized Items is 0.744). The eight domains also had a good overall internal consistency (Cronbach alpha = 0.780). The internal consistency (expressed by the Cronbach alpha) of most domains was over 0.6. Only one domain, that of emergency training and drills, had an internal consistency which fell under 0.6 (0.406).

## 4. Discussion

### 4.1. Significance

The concept of hospital resilience provides a comprehensive approach to improving a hospital’s ability to withstand the impact of a disaster, and to reduce the mortality and mobility associated with disasters through efficient health responses. In this study, the principal factor analysis was conducted to provide a four-factor solution for hospital resilience.

The significance of this study is three-fold. Firstly, the proposed framework launches an important discussion regarding the conceptual understanding of what constitutes hospital disaster resilience. Departing from the majority of existing research, that focuses on only one or two aspects of a hospital’s disaster ability (such as preparedness), the framework captures the principal and comprehensive components to depict a hospital’s overall ability to cope with disasters. It is proposed that a multidimensional measurement is more reliable and comprehensive for measuring a hospital’s overall resilience than examining its disaster capability through only one aspect or a limited number of aspects. Thus, the proposed framework measures preparedness before disasters, and also describes the underlying elements of disaster functions and health outcomes, *i.e.*, hospital emergency medical response, recovery and adaptation strategies.

Secondly, the results of the factor analysis are encouraging. It yielded factors largely consistent with the conceptualization of hospital resilience and the construction of its primary domains. It generated a four-factor structure of hospital resilience, namely: emergency medical response capability (F_1_), disaster management mechanisms (F_2_), hospital infrastructural safety (F_3_), and disaster resources (F_4_). It seems that the four factors move from the micro level to the macro level. The F_1_ was at the ground level and was about immediate “doing”, which encompasses emergency medicine capability, surge capacity, and staff capability; F_2_ was relevant to management, plans and cooperation. F_2_ was not immediate “doing”, but rather, it was about having policies and procedures in place that can be enacted at the right time. F_3_ and F_4_ were concerned with infrastructures and resources which involve macro-level planning for safety, such as standards, procedure, and disaster resources.

Finally, the four-factor structure offers a way of modelling the overall level of hospital resilience and the level of four factors independently. There are two ways of modelling: one way is to use the self-assessment scales for categorizing the level of hospital resilience. It uses the questionnaire as a simple checklist for hospital self-evaluation. It can provide an overall score, as well as provide scores on particular areas of resilience. Thus, it can enable hospitals to drill down to identify areas for improvement and can be used as a basis for measuring improvement over time. Further, the scales can be adjusted and reconstructed with more items being added into the questionnaire for future studies. Even if the highest scores (*i.e.*, total number of questions) are different, the same classification criteria (25%, 75%) can still be used for the classification of the levels [47]. Alternatively, the regression modelling of different weights for each factor can be used. The modelling also has regional application as it enables regions or provinces to drill down into the resilience of their hospitals. The scores can provide a comparison of hospital resilience for hospitals of similar size and function within and across regions. Thus, the regions and provinces can build capacity in a targeted and coordinated way.

The strength of this study is that the preliminary evaluation framework and the derived questionnaire were developed from a systematic approach (using an international literature review, the Modified- Delphi Method in China, and the empirical data). The internal consistency of the framework and questionnaire was relatively high; its construct validity was validated to some extent. This preliminary framework can provide a foundation for further similar studies or evaluations.

Furthermore, according to the experts’ opinions in the Delphi study, except for the hospital routine treatment, tertiary hospitals are also expected to have outreach functions, and to cope with major disasters (e.g., earthquakes, bioterrorism, and epidemics) that can cause mass casualties on-site. Hospital resilience was an essential element of resilient communities, therefore, hospitals need to provide immediate healthcare services to the affected communities [49]. However, critical healthcare services include both patient transport into hospital and the bringing of care to the patient on site [50]. Thus, during major disasters, tertiary hospitals need to be capable of providing expert teams, equipment and medical care services into the field, for on-site medical rescue, as well as to support the treatment provided by the local hospitals for a large number of patients [51,52]. At present, such functions are commissioned by the local government from the local tertiary hospitals, in order to build a district coordinated medical response network for major disasters [52].

### 4.2. Model Justification

The weights of these four factors were calculated using factor analysis. However, the calculated weights needed to be justified from the resilience concept and the actual resilience status of the hospital. Hospital resilience is a comprehensive concept; it includes structural components (e.g., facility and infrastructural safety), non-structural components (e.g., network, staff, equipment, medication), emergency healthcare service components (e.g., medical response and treatment, continuity of medical service, surge capacity, training and drills), and disaster management (e.g., plan and procedure, command, cooperation, crisis communication, recovery and adaptation strategies) [10,12,53]. Thus, the eight primary domains and the extracted four factors complied with the concept, and made intuitive sense.

Among the four factors, hospital medical response capability (F_1_) was identified as the main component factor, as it was relevant to the emergency healthcare performance and was health outcome-oriented in times of disasters. F_1_ was merged with three other domains (*i.e.*, emergency service capability and surge capacity, emergency staff capability, disaster training and drills); the combination made intuitive sense. The domain of emergency service capability (e.g., medical equipment, on-site rescue, triage of patients, and priority of functions) and surge capacity (*i.e.*, surge beds, supplies and staff) were essential for the hospital medical performance during disasters. The presence of key staff, and ensuring that they were suitably trained and ready, also impacted upon the hospital’s disaster performance [27,44]. Hence, F_1_ was about the “doing” capacity at the ground level, and linked directly to the hospital medical care functions during disasters.

Hospital disaster management mechanisms (F_2_) were the second most highly weighted factor, and it consisted of disaster plans, command and control system, cooperation, communication, recovery and adaptation strategies. The F_2_ reflects the basic disaster management system, which refers to the resourceful strategies (adaptive flexibility) that can be used to improve hospital operational functions and, thus, to achieve hospital disaster resilience. F_2_ was used to efficiently recover affected hospital essential functions, using a wide variety of disaster management approaches including “various disaster plans”, “command and control system”, “communication protocols”, “cooperation with community facilities”, “strategies to prioritize and maintain essential health functions”, “strategies to share and manage emergency supplies and staff”, and “recovery and adaptation strategies after disasters” (these illustrated indicators are presented in Table 2). Thus, F_1_ and F_2_ were the key components influencing the speed with which the recovery of a hospital’s essential functions were achieved (rapidity); consequently, they should be the core component of hospital disaster resilience [21].

The factors of hospital infrastructural safety (F_3_) and hospital disaster resources (F_4_) weighted less highly. Physical infrastructure safety was found to have a significant effect on maintaining hospital functionality during various disasters (e.g., earthquakes, floods, fires and epidemics), and the redundancies of network can increase hospital resilience [54,55]. F_3_ in this study refers to two issues: the infrastructure (or procedures) of a hospital that were compliant with the construction standards to guarantee hospital internal safety; and using the redundancies of utility networks (e.g., equipment, communication, power, and water pumping) to continue hospital functions during disasters. It may be controversial that hospital infrastructure did not weight more prominently. After all, hospital infrastructure is essential to resilience as surge capacity and other response solutions could not occur in the face of infrastructure collapse [54,55,56]. However, the evaluation framework of hospital resilience in this context was meant to be used by hospital managers and governmental administrators. Thus the engineering perspective, especially of infrastructure nonstructural components (e.g., transportation systems, elevators, ceilings and utility system) [12,54,55,56] was likely to have been understated, as more items focused on the perspective of disaster management, emergency medicine capability and capacity.

F_4_ was used to reflect the critical resources, including medications and supplies that were used to achieve health function continuity. The stockpile of these resources should comply with the hospital emergency medicine catalog and emergency resources reservation plan that were issued by the local health department. The critical supplies included food, water, hand hygiene, stretchers, wheelchairs, ventilators, IV pumps, tourniquets, personal protective equipment, *etc.*, while the critical medications included antimicrobial agents, cardiac medications, insulin, anti-hypertensive agents, IV fluids, *etc.* [57]. Although different levels of hospitals may have a wide variation in disaster resources and stockpiles, hospitals should be prepared with self-sustained supplies and medications for a disaster for 2–3 days, regardless of the level of structural damage [57,58]. Broadly based strategies for resources logistics management (e.g., share with other facilities, sign with pharmacy companies, priority distribution) should also be applied to maintain and surge these critical resources. In summary, F_3_ and F_4_ together can be used to reflect a hospital’s robustness (the extent to which function is maintained) to withstand disasters, initially without external assistance.

### 4.3. Limitations

There are several limitations to this study. Firstly, the framework was tested on a relatively small sample (*n* = 41) of tertiary hospitals in one province in China, which restricts the generalizability of the findings. Thus, the results may not be directly transferable to hospitals in other areas or countries, or to smaller facilities. While the nature of this work can be considered as preliminary or exploration, it is appropriate to test the framework in a region or province, prior to a larger roll-out, such as a national study. Hence, the proposed framework needs to be adapted into other contexts (e.g., other areas or countries) for further validation. Secondly, due to ethical considerations, the surveyed hospitals were guaranteed anonymity. It was, therefore, not possible to compare and validate their resilience score (see Table 5) with their real status of emergency medicine and disaster preparedness. Thirdly, the questionnaire tends to measure hospital resilience from different dimensions, with each dimension contributing different weights to the overall resilience. The weight was calculated through the empirical data, so it would be beneficial to be validated further with experts’ opinions and additional empirical studies. Finally, the framework was derived from a systematic review and Delphi study in China. Thus, the indicators in the framework may not capture all possible indicators, and its generalizability is limited. With the addition of further expert perspectives (e.g., engineering, emergency medicine experts from other countries), as well as newer information that became available since the conduct of the survey, more items may be added into the construct and retested for validation through ongoing research. Additionally, the self-evaluation scales and the score model could be adjusted accordingly. Despite the limitations, the framework can be regarded as a checklist to evaluate key indicators of hospital vulnerability and to identify priority practices that could better prepare the facilities for future disasters. The methodological framework and some of the agreed indicators may inform the development of indicators of hospital resilience in other countries or areas.

## 5. Conclusions

The comprehensive framework provides a way to conceptualize hospital resilience, and a foundation for developing a user-friendly instrument for measuring it. A four-factor structure of hospital resilience was identified. The reliability and construct validity of this framework was tested. Although additional work is still needed, the findings provide a basic framework and foundation for future research.

## Supplementary Files

Supplementary File 1 Supplementary Information (PDF, 259 KB)
