# Supplementary material for: Validation of a Framework for Measuring Hospital Disaster Resilience Using Factor Analysis
Source: Int J Environ Res Public Health. 2014 Jun 18;11(6):6335–53. doi: 10.3390/ijerph110606335 (PMC4078582; doi:10.3390/ijerph110606335)
Supplement: Supplementary File 1 — Supplementary Information (PDF, 259 KB) [file ijerph-11-06335-s001.pdf]

Validation of a Framework for Measuring Hospital Disaster Resilience Using Factor Analysis

---

Questionnaire (English Translation Version)

Questionnaire for assessment of disaster resilience capability in tertiary hospitals of Shandong Province.

Introduction Note (Please Read First before Filling the Form)

1. Public emergencies and disasters in the questionnaire refer to events that suddenly happened and can cause serious impact to the society, which require emergency measures to be taken. These events include natural disasters (e.g., earthquakes, floods), disasters arising from accidents (e.g., transportation incidents, environmental pollution), public health incidents (e.g., emerging infectious diseases, food poisoning) and public security incidents (e.g., terrorism).

2. Fill method: There are two types of questions: (1) choice questions: Unless it is marked with "this question has multiple choices", these questions are single choice (*i.e.*, select one answer), please choose the option that can meet the hospital's situation, which is after each question (e.g., (1) (2))/by tick "√"; (2) fill in the blank: please fill out the relevant data/content into the underscored place after each question. Note the logical question to jump to the next question.

3. Please be sure to complete the form within the required time. After its completion, please report them simultaneously both via e-mail and postal.

Thank you for your assistance and support to the investigation!

****

Hospital name:_______________fill date:_____month_____date 2012

The informant (signature)_____Audit dean (signature)_____Official seal:(stamp)

A. The General Situation Relevant to Emergency Medicine

1. Hospital level: Tertiary________(e.g., A, B, C).
2. Hospital location (in details to the county, city, and district):
3. Hospital attribution: (1) government health administrative office (2) industry hospital (3) others
4. Hospital type: (1) general hospital (2) specialty hospital (3) others
5. Hospital mission: (1) be assigned regional disaster rescue mission (2) not-assigned the mission
6. The total number of hospital beds__sheets;
    6.1. Among them, the number of licensed beds______sheets;
    6.2. The number of intensive care beds (ICU)________sheets;
    6.3. The number of beds in emergency department (ED) or emergency care centre ____sheets;

6.4. The number of monitored beds______?
6.5. The number of paediatric beds______?

Signature of the Responsible Person______________ (from department:____________)

B. Hospital Safety Standard and Procedures

7. Whether the hospital establishes a syndromic surveillance and early warning system for public health emergencies? (1) Yes (2) No;
   7.1. The syndromic that need to be surveillance, report and early warning include:
   7.2. Whether develop and require that physicians on duty report any suspicious cases to the hospitals' presidents? (1) Yes (2) No;
8. Whether the hospital has direct online reporting system of surveillance information and suspicious symptoms? (1) Yes (2) No;
   8.1. Whether the hospital could analyze surveillance data regularly and share this system with the local health authority? (1) Yes (2) No;
9. Is there any pre-event evaluation of types and impact of the potential risks to hospitals in its location? (1) Yes (2) No;
   9.1. When the disaster occurred, within the hospital is there any hazards identification system for different types of risks? (1) Yes (2) No;
   9.2. When there is hospital internal risk, are there any strategies for hospitals to evacuate and protect existing patients? (1) Yes (2) No;
10. Is there any evaluation of the safety standards of hospital's critical infrastructures? (e.g., construction safety standard of resistance to earthquakes, fires, floods and infectious diseases). (1) Yes (2) No;
    10.1. If yes, were the critical infrastructures (e.g., buildings, operation rooms, ICU) built to meet or excess the local criteria of resistance to earthquake? (1) Yes (2) No;
    10.2. If yes, were the critical infrastructures built in a higher position in the area to prevent floods? (1) Yes (2) No;
    10.3. If yes, was the critical medical equipment located in a higher level of the building to prevent floods? (1) Yes (e.g., the first floor with a higher location, the second floor) (2) No (e.g., underground, the first floor with a lower location);
    10.4. If yes, is there any consideration of the safety standard for the risk of fire? (1) Yes (2) No;
    10.5. If yes, is there any consideration of using isolated pathways and designated areas for infectious diseases within the hospital? (1) Yes (2) No;
11. When disaster occurred, are there any alternative emergency energy and facilities for backup (e.g., power, oxygen and water pumping, telecommunication system)? (1) Yes (2) No;

Signature of the Responsible Person______________ (from department:____________)

C. Emergency Command, Communication and Cooperation System

12. Is there any disaster committee or disaster group (includes positions from multidisciplinary departments) within hospital that is responsible for public emergencies? (1) Yes (2) No;
    12.1. Is there any official document that has been used to establish hospital disaster committee or disaster group? (1) Yes (2) No;
13. Is there any department (has regular contact with the chiefs in multidisciplinary departments) within the hospital that has been assigned responsibility for the work relevant to emergencies? (1) Yes (2) No;
    13.1. Is there any official document that has been used to assign emergency relevant work to this department? (1) Yes (2) No;
14. Is there any coordinating meeting with key staff from different hospital departments during emergencies? (1) Yes (2) No;
15. Has the hospital attended regional coordinating meeting with other emergency departments during emergencies, such as CDC (Center for Disease Prevention and Control), pre-hospital emergency system, healthcare facilities, blood and resource center, and local government? (1) Yes (2) No
16. Is there any crisis communication protocol that can be used for communication during emergencies (e.g., communication with other community departments, the public and mass)? (1) Yes (2) No

Signature of the Responsible Person_____________ (from department:___________)

D. Hospital Disaster Plans

17. Is there any general disaster plan and relevant document in place for preparedness of public emergencies? (1) Yes (2) No;
    17.1. Please illustrate the document name of the disaster plan?
18. Are there any specific disaster plans based on the specific requirements of a single hazard, such as infectious diseases, internal medical accidents, public health emergencies, natural disasters, bio-terrorism and nuclear terrorism, and others? (1) Yes (2) No;
    18.1. Please illustrate the document name of the specific disaster plans?
19. Is there any protocol to initiate the plan, so as to guarantee the hospital be in place to face emergencies immediately, (*i.e.*, guarantee staff, equipment and resources are in place immediately)? (1) Yes (2) No;
20. From the experience of the hospital dealing with the past public emergencies (*i.e.*, mass casualty incident, disasters, pandemics), whether the hospital could operate in accordance with the disaster plan during emergencies? (1) Yes (2) No;
21. Is there any classification response system to cope with different levels and different phases of events? (1) Yes (2) No;
22. Is there any evaluation and revision of the disaster plans in the last two years? (1) Yes (2) No;
23. Is there any dissemination of the content of disaster plans to the key staff (e.g., through regular meetings or training)? (1) Yes (2) No;

Signature of the Responsible Person_____________ (from department:___________)

E. Emergency Stockpiles and Logistics Management

24. Are there any stockpiles of various types of emergency drugs within hospitals (e.g., antimicrobial agents, cardiac medications, insulin, anti-hypertensive agents, IV fluids)? (1) Yes (2) No;
    24.1. If yes, please illustrate the type names and quantities of these stockpiled drugs?
25. Dose the facility have a means of real-time inventory and tracking of the following resources: including food, water, hand hygiene, stretcher, wheelchair, ventilators (adult), ventilators (pediatric), IV pumps, IV poles, and tourniquet? (1) Yes (2) No;
    25.1. If yes, please illustrate the type names and quantities of the stockpiled materials?
    25.2. Whether the hospital has the following personal protective equipment (PPE) *(multi- choices)*?
        (1) biohazard protective suits; (2) goggles; (3) ventilator; (4) N95 Masks
26. When there is mass casualty incident, whether the hospital could be able to load and deliver emergency drugs for on-site rescue? (1) Yes (2) No;
    26.1. If yes, please illustrate the names and quantities of the emergency drugs that could be loaded and delivered for on-site rescue?
27. Whether the hospital has the following strategies for management of drugs and materials?

	Strategies for Management of Drugs and Materials?	Yes	No
27.1	Drug-distribution plans to identify distribution priority of drugs during crisis	□	□
27.2	Signed contracts with emergency drug-supplies to provide drugs during emergencies	□	□
27.3	Signed Memorandum of Understandings (MOUs) with other hospitals to share emergency drugs during emergencies	□	□
27.4	Share and obtain these materials from relevant industries during emergencies	□	□
27.5	Share and obtain these materials from other hospitals during emergencies	□	□
27.6	Others: (please illustrate)	□	□

Signature of the Responsible Person_____________ (from department:___________)

F. Emergency Staff

28. The composition of hospital experts group (refer to those members within the hospital that are involved in development of the emergency plans and specific emergency medical treatment)
    (1) General surgical treatment____ persons, including senior ____persons;
    (2) General medical treatment____persons, including senior____persons;
    (3) Neurosurgery ____persons, including senior____persons;
    (4) Bone surgery____persons, including senior____persons;
    (5) Burn ____persons, including senior____persons;
    (6) Psychological ____persons, including senior____persons;
    (7) Emergency Department ____persons, including senior____persons;
    (8) ICU ____persons, including senior____persons;
    (9) Nosocomial infections___persons, including senior____persons;
    (10) Total experts___persons, including senior____persons
29. Is there any emergency staff that could be dispatched during disasters for the on-site rescue? (1) Yes (2) No;

If yes, please fill the composition (*i.e.*, specialty and numbers) of emergency staff that can be dispatched

(1) General surgical doctors,____persons, general surgical nurse____persons ;

(2) Therapeutic,____persons, general medical nurse____persons;

(3) Neurosurgeon doctors____persons, neurosurgery nurses____persons;

(4) Orthopedic surgeon doctors____persons, orthopedic surgeon nurse____persons;

(5) Burn treatment doctors____persons, burn treatment nurses____persons;

(6) Psychological doctors____persons;

(7) Emergency department doctors____persons, emergency nurses____persons;

(8) ICU doctors____persons, ICU nurses____persons;

(9) Infections control doctors____persons, infections control nurses____persons;

(10) Manager people____persons, including managers____persons, information people persons; logistics____persons; and other relevant personnel____persons

(11)Total doctors ____persons, total nurses____persons;

30. Whether the hospital has the following incentive and protective strategies for management of emergency staff?

**Incentive and Protective Strategies for Emergency Staff?**	**Yes**	**No**
30.1 Incentive strategies for emergency staff	□	□
30.2 Vaccination for emergency staff and their family members	□	□
30.3 Insurance for emergency staff	□	□
30.4 Others (please illustrate):	□	□

31. Whether the hospital has the following incentive strategies? (Can be multiple-choices) (1) increase the salary; (2) increase the vacation; (3) priority for hiring and position promotion; (4) Honours; (5) issue the grant

Signature of the responsible person_____________ (from department:___________)

G. Emergency Services and Surge Capability

Hospital Treatment (Refer to the Hospital Daily Internal Treatment Capability)

32. The total number of hospital beds___sheets;

32.1. Among them, the number of isolation beds________?

32.2. The number of orthopedic beds________?

32.3. The number of suspension bed for burns________?

32.4. The number of emancipated bed________?

32.5. The number of surgery rooms________?

32.6. The number of hyperbaric oxygen chambers________?

33. The number of Intensive Care Unit (ICU)____?

33.1. Whether the hospital has breathing machines in ICU? (1) Yes (2) No;

33.2. Whether the hospital has vital signs monitors in ICU? (1) Yes (2) No;

33.3. Whether the hospital has defibrillator machines in ICU? (1) Yes (2) No;

33.4. Whether the hospital has cardiac resuscitation devices in ICU? (1) Yes (2) No;
33.5. Whether the hospital has CRRT devices in ICU? (1) Yes (2) No;

34. Whether the hospital has internal capacity (e.g., space, beds and experts) for treating mass casualty of incidents (*i.e.*, here mass casualty treatment capacity refers to each hospital is to assess itself on its capacity to accept at least 30 patients of the same disease within a short period)? (1) Yes (2) No;
    34.1. Whether the hospital has capacity (e.g., space, beds and experts) for treating general mass casualty of trauma? (1) Yes (2) No;
    34.2. Whether the hospital has capacity (e.g., space, beds and experts) for treating mass casualty of infectious diseases? (1) Yes (2) No;
    34.3. Whether the hospital has capacity (e.g., space, beds and experts) for treating mass casualty of blast injury, gunshot wounds and crush injury? (1) Yes (2) No;
    34.4. Whether the hospital has capacity (e.g., space, beds and experts) for treating mass casualty of acute chemical poisoning? (1) Yes (2) No;
    34.5. Whether the hospital has capacity (e.g., space, beds and experts) for treating mass casualty of radiation issues? (1) Yes (2) No;

Hospital Surge Capacity (Refer to the Hospital Medical Surge Capability Response to the Disasters)

35. When disaster occurs, is there any internal evaluation mechanism for rapid assessment of the available emergency resources and the disaster losses? (*i.e.*, manpower, equipment, number of emergency beds)? (1) Yes (2) No;
36. Are there any prepared spaces and conditions (e.g., electricity, oxygen, water, heat) in place to temporary surge numbers of emergency beds? (1) Yes (2) No;
    36.1. If yes, are there any plans and work procedures for surging emergency beds? (1) Yes (2) No;
    36.2. The maximum surge capacity of emergency beds are____sheets (within 24 h)
37. Are there any procedures and strategies to vacate part of the occupied emergency beds for treating the sick and wounded from emergency events according to the requirement? (1) Yes (2) No;
    37.1. When emergency occurs, according to the instruction from health administrative departments and the actual situation of admitted patients, within 24 h, the hospital can maximum vacate the occupied emergency beds of_______sheets?
38. Whether the hospital has a wide variety of flexible procedures for surging beds capacity when it faces the emergencies?

Surge Procedures for Emergency Beds	Yes	No
38.1 cancellation of elective admissions	□	□
38.2 early discharge of patients	□	□
38.3 transfer patients to primary health care and other facilities	□	□
38.4 others (please illustrate):	□	□

39. Whether the hospital has a wide variety of flexible procedures for surging emergency staff capacity when it faces the emergencies?

Surge Procedures for Emergency Staff	Yes	No
39.1 training and transferring non-critical care staff to support critical care	□	□
39.2 recalling all the off-work staff back to work	□	□
39.3 rehiring retired staff	□	□
39.4 sharing staff from other hospitals	□	□
39.5 using volunteers or temporary employers	□	□
39.6 suppling living places for staff		
39.7 others (please illustrate):	□	□

40. Are there any mass-casualty triage procedures for admission of patients who require urgent critical care during disasters? (1) Yes (2) No;

Hospital On-site Rescue (Refer to Patient Transport and Bringing Care to the Patient On-site)

41. Whether the hospital has its own ambulances? (1) Yes (2) No;
    41.1. If yes, are there any ward-type ambulances? (1) Yes (2) No;
    41.2. If yes, are there any negative pressure isolation ambulances? (1) Yes (2) No;
    41.3. Whether the hospital has on-site command vehicle? (1) Yes (2) No;
42. Whether the hospital has rescue helicopters and access to a helicopter landing pad? (1) Yes (2) No;
43. Is there any on-site communication equipment for data transmission, video-audio connection, and remote consultation? (1) Yes (2) No;
44. Whether the hospital could organise an independent rescue team that is equipped with emergency package of supplies (e.g., clean water, food, tent, and lighting) for living 3 days (the teams include those health administrative departments or other departments assigned to construct based on the hospital)? (1) Yes (2) No;
    44.1. If yes, please illustrate the number of staff for the rescue teams, and their departments and specialty:
    44.2. If yes, are the rescue teams equipped with portable medical equipment equipped (e.g., portable breathing machine, ECG monitoring machine, and the X-ray machine? (1) Yes (2) No;
45. Whether the hospital has 'portable hospital' or the capability to support field surgery, and other critical care in the field, which is similar to the function of ICU (using vehicles which are equipped with beds and portable medical equipment)? (1) Yes (2) No;

Signature of the responsible person_____________ (from department:___________)

H. Emergency Training and Drills

46. Are there any disaster or emergency training programs? (1) Yes (2) No;
47. Are there any disaster or emergency drills? (1) Yes (2) No;
48. If yes, are there any disaster training programs and drills treating the following emergency types respectively during 2011–2012?

Types for Disaster Training Programs and Drills	Has Training	Has Drills
48.1 infectious disease （e.g., SARS, H_7N_9）	□	□
48.2 mass casualty incidents (e.g., natural disasters)	□	□
48.3 career poising and food poising	□	□
48.4 bio-terrorism and nuclear terrorism	□	□

49. Are there any disaster training curriculums? (1) Yes (2) No;
    49.1. If yes, were the training curriculums updated regularly? (1) Yes (2) No;
50. In 2011–2012, is there any emergency training including the following content?

Content of Emergency Trainings	Yes	No
50.1 basic skills for the treatment of trauma	□	□
50.2 cardiopulmonary resuscitation	□	□
50.3 trachea cannulation	□	□
50.4 transfer of casualties	□	□
50.5 triage	□	□
50.6 disaster management	□	□
50.7 others (please illustrate):	□	□

51. Is there any emergency or disaster training regularly every two years? (1) Yes (2) No;
52. Are there any disaster drills regularly every two years? (1) Yes (2) No;
53. Are there any drills the hospital cooperating with all the other emergency facilities of the community (such as police, fire, water, and transport sectors)? (1) Yes (2) No;

Signature of the Responsible Person_____________ (from department:___________)

I. Recovery and Adaptation Strategies

54. Is there any special department that would be assigned to be responsible for the work relevant to recovery and reconstruction? (1) Yes (2) No;
55. Are there any specific channels of investing money, transferring staff, and purchasing equipment for recovery phases after the event? (1) Yes (2) No;
56. Whether the hospital has been involved or would be involved in the health related work of the affected communities?

Be Involved in the Health Related Work of the Affected Communities?	Yes	No
56.1 be involved in the design of the recovery strategies for the community	□	□
56.2 health status evaluation of the population in the community	□	□
56.3 health social or health interventions to the community	□	□
56.4 rehabilitation for the victims	□	□
56.5 psychological consultation for relevant people	□	□
56.6 others (please illustrate):	□	□

57. Is there any mechanism of after-event evaluation report? (1) Yes (2) No; If yes, is the following content need to be included in the evaluation report?

**Evaluation Content**	**Yes**	**No**
57.1 local high risks re-evaluation	□	□
57.2 hospital capability evaluation	□	□
57.3 hospital vulnerability evaluation	□	□
57.4 experience and lessons that have been learned	□	□
57.5 others (please illustrate):	□	□

58. Are there any adaptation strategies after disasters (maybe involved in the disaster evaluation report) designed for hospitals to better cope with future disasters? (1) Yes (2) No;

Signature of the Responsible Person_____________ (from department:___________)

© 2014 by the authors; licensee MDPI, Basel, Switzerland. This article is an open access article distributed under the terms and conditions of the Creative Commons Attribution license (http://creativecommons.org/licenses/by/3.0/).
